# Pax2/8 act redundantly to specify glycinergic and GABAergic fates of multiple spinal interneurons

**DOI:** 10.1016/j.ydbio.2008.08.009

**Published:** 2008-11-01

**Authors:** Manuel F. Batista, Katharine E. Lewis

**Affiliations:** Department of Physiology, Development and Neuroscience, Anatomy Building, Downing Street, Cambridge CB2 3DY, UK

**Keywords:** Pax2, Pax8, GABA, Glycine, Interneuron, Spinal cord, Zebrafish, Pax2a, Pax2b, Neurotransmitter

## Abstract

The spinal cord contains several distinct classes of neurons but it is still unclear how many of the functional characteristics of these cells are specified. One of the most crucial functional characteristics of a neuron is its neurotransmitter fate. In this paper, we show that in zebrafish most glycinergic and many GABAergic spinal interneurons express Pax2a, Pax2b and Pax8 and that these transcription factors are redundantly required for the neurotransmitter fates of many of these cells. We also demonstrate that the function of these Pax2/8 transcription factors is very specific: in embryos in which Pax2a, Pax2b and Pax8 are simultaneously knocked-down, many neurons lose their glycinergic and/or GABAergic characteristics, but they do not become glutamatergic or cholinergic and their soma morphologies and axon trajectories are unchanged. In mouse, Pax2 is required for correct specification of GABAergic interneurons in the dorsal horn, but it is not required for the neurotransmitter fates of other Pax2-expressing spinal neurons. Our results suggest that this is probably due to redundancy with Pax8 and that the function of Pax2/8 in specifying GABAergic and glycinergic neuronal fates is much broader than was previously appreciated and is highly conserved between different vertebrates.

## Introduction

The spinal cord is a crucial part of the vertebrate central nervous system as its neuronal circuitry controls movements and receives sensory inputs from the trunk and limbs. For this circuitry to function, different classes of neurons have to be correctly specified in the developing embryo. These cells form at specific dorsal/ventral locations in the spinal cord and express particular combinations of transcription factors (Lewis, 2006 and references therein). Currently, we know a lot about how these cells become molecularly-distinct from each other and express particular combinations of transcription factors (Lewis, 2006 and references therein) but we know a lot less about how the expression of particular transcription factors relates to the development of specific functional neuronal characteristics.

One crucial identifying characteristic of a neuron is the neurotransmitter(s) that it releases. How this is determined is still not very well understood and it is unclear whether similar or different mechanisms operate in distinct classes of neurons. Currently, specification of neurotransmitter fate is best understood in the mouse dorsal spinal cord, where Lbx1, Ptf1a, Lhx1 and Lhx5 transcription factors act upstream of Pax2 to specify GABAergic cells and Tlx1/3 antagonise the effects of Lbx1 to specify glutamatergic cells (Cheng et al., 2004, 2005; Glasgow et al., 2005; Pillai et al., 2007). In the mouse, Pax2 is also expressed in more ventral interneurons, but the neurotransmitter fates of these cells are unchanged in Pax2 knock-out mice (Cheng et al., 2004; Pillai et al., 2007), suggesting that Pax2 has distinct functions in different interneurons.

In the zebrafish, *Danio rerio*, there are two *pax2* genes (*pax2a* and *pax2b*) and these both have a very similar spinal cord expression pattern to the highly-related *pax* gene, *pax8* (Figs. 1A, B and E; Pfeffer et al., 1998). In this paper, we show that in zebrafish the vast majority of glycinergic spinal interneurons express Pax2/8 as do most GABAergic neurons within the spinal cord region where *pax2/8* genes are expressed. When we knock-down just Pax2a, Pax2b or Pax8, there is very little effect on these glycinergic and GABAergic interneurons, but if we knock-down all three of these Pax2/8 proteins many interneurons lose their glycinergic and/or GABAergic fates, including the vast majority of CiAs, which are the most ventral population of Pax2/8 expressing spinal cells. We also establish that this function of Pax2/8 in spinal interneurons is very specific: cells lose their glycinergic and/or GABAergic fates in triple knock-down embryos, but they do not become glutamatergic or cholinergic. In addition, the soma morphozlogies and axon trajectories of CiAs are unchanged.

Our results suggest that the limited phenotype in the *Pax2* mutant mice may be due to redundancy with Pax8 and consistent with this, in these mouse mutants Pax8 continues to be expressed in the ventral spinal cord, but it is lost from the Pax2-expressing cells that migrate into the dorsal horn (Pillai et al., 2007). This suggests that the function of Pax2/8 in specifying glycinergic and GABAergic neuronal fates is much broader than was previously appreciated and is highly conserved between different vertebrates.

## Materials and methods

### Zebrafish lines

Zebrafish (*D*. *rerio*) embryos were obtained from wild-type (AB, TL, or AB/TL hybrids) or Tg(*pax2a*:GFP) adults (Picker et al., 2002) or identified carriers heterozygous for *noi*^*tu29a*^, a null allele of *pax2a* (Lun and Brand, 1998). Embryos were staged by hours post-fertilisation at 28.5 °C (h) and/or appropriate morphological criteria as in Kimmel et al. (1995). *noi* mutants were identified by their lack of a midbrain/hindbrain boundary (Brand et al., 1996; Lun and Brand, 1998).

### *In situ* hybridisation and immunohistochemistry

*In situ* hybridisation was performed as in Concordet et al. (1996). For double *in situ* hybridisation, embryos were treated with Signal Enhancer (Invitrogen) before antibody incubation. Mouse-Anti-Dig (1/5000, Jackson ImmunoResearch) and Rabbit-Anti-Flu (1/2500, Roche) were detected with Invitrogen Tyramide kits #12 (488) and #5 (594). For *in situ* hybridisation and immunohistochemistry double stainings, Rabbit-Anti-GFP (1/1000) from Molecular Probes or Rabbit-Anti-Pax2 (1/300) from BabCO was revealed with Alexa-Fluor Goat-Anti-Rabbit 488 (1/1000) from Molecular Probes. RNA was detected with Invitrogen Tyramide kit #5. Double immunohistochemistry experiments to simultaneously detect Pax2 and GFP or Eng1b and GFP utilised Mouse-Anti-GFP (1/300) from Molecular Probes (catalogue # A11121). Eng1b antibody was kindly provided by A. Joyner (Skirball Institute, New York). This antibody (anti-Enhb-1; rabbit polyclonal) was generated against the mouse En2 homeodomain and detects both En1 and En2 in mammals (Davis et al., 1991) but only Eng1b in zebrafish (Higashijima et al., 2004b).

To determine neurotransmitter phenotypes we used *in situ* hybridisation for genes that encode proteins that transport or synthesise specific neurotransmitters. *glyt2a* and *glyt2b* (glycinergic markers) encode for glycine transporters necessary for glycine reuptake and transport across the plasma membrane; *gad65*, *gad67a* and *gad67b* (GABAergic markers) encode for a glutamic acid decarboxylase, necessary for the synthesis of GABA from glutamate and *vglut2*.*1*, *vglut 2*.*2a* and *vglut 2*.*2b* (glutamatergic markers) encode proteins responsible for transporting glutamate to the synapse. In all of these cases, a mix of equal concentrations of the relevant probes was used (Higashijima et al., 2004a,c). *Choline acetyltransferase* (*chat*) encodes for an enzyme that catalyzes the synthesis of acetylcholine (Yokogawa et al., 2007).

Other probes were *pax2a*, *pax2b*, *pax8*, *pax5* (Pfeffer et al., 1998), *p53* (Robu et al., 2007) and *eng1b* (1.3 KB encompassing the ORF, a kind gift from Drs. Kikuchi and Westerfield at the University of Oregon).

Photographs were taken using a Zeiss Axio Imager M1 (DIC images) or a Leica TS SP2 confocal (fluorescent images) microscope and processed using Adobe Photoshop. All fluorescent images, with the exception of Figs. 2H–J and Figs. 4A'– E' and I' are projections of multiple optical sections performed in Image J (Abramoff et al., 2004).

### Morpholino injections and controls

Morpholino antisense oligonucleotides (MOs) were injected into 1–2 cell embryos from a cross of identified carriers heterozygous for *noi*^*tu29a*^. Therefore, ∼ 25% of injected embryos lacked Pax2a. All of these morpholinos have been used successfully in previous studies. The *pax2b* MO blocks translation and is GGTCTGCCTTACAGTGAATATCCAT (Bricaud and Collazo, 2006; Mackereth et al., 2005; Millimaki et al., 2007); the *pax8* MOs block splicing and a combination of E5/15(TTTCTGCACTCACTGTCATCGTGTC) and E9/19(ACCGGCGGCAGCTCACCTGATACCA) (Hans et al., 2004) were used.

MOs were initially injected at concentrations of 1 mg/ml, 1.5 mg/ml and 2 mg/ml each. At 1.5 mg/ml and 2 mg/ml the phenotype was more severe than at 1 mg/ml (Supp. Data Figs. 4B and B'). However, at 2 mg/ml some embryos were morphologically disturbed (they had twisted axis and wavy notochords). Therefore, in all of the experiments presented in this paper, with the exception of Supplementary Data Fig. 1B, MOs were injected at 1.5 mg/ml each. At this concentration, triple-knock-down embryos (*noi* mutants injected with *pax2b* and *pax8* MOs) completely lost expression of *pax8* RNA and Pax2 protein (Figs. 1F and 3I), suggesting that Pax2a, Pax2b and Pax8 were fully knocked-down. To further confirm that the *pax8* splice-blocking MOs were working we examined *pax8* expression in embryos injected with a lower concentration of morpholinos (1 mg/ml). In this case, *pax8* RNA was localised in the nucleus, indicating that splicing of *pax8* RNA was blocked (Yan et al., 2002; cf.Supp. Data Figs. 1A and B).

To rule out the possibility that the phenotype observed in morpholino injected embryos was due to either specific or non-specific toxicity or cell death we examined *p53* expression in our injected embryos at 36 h. Non-specific cell death due to MO toxicity usually causes an upregulation of *p53* expression (Robu et al., 2007). However, there was no expression of *p53* in our injected embryos (36 h; *n* = 60; Supp. Data Fig. 1H). In addition, we demonstrated that *pax2/8*-expressing cells still form in morpholino injected embryos using an *in situ* hybridisation for *pax2a* and *pax2b* (Figs. 1D and K). We saw no significant changes in the number of cells expressing these genes in triple knock-down embryos compared to wild-type embryos. Therefore, the cells still form and the reduced number of glycinergic and GABAergic cells in triple knock-down embryos is not due to a reduction in the number of *pax2/8*-expressing cells. Finally, we also showed that CiAs, which are a specific subset of *pax2/8*-expressing cells, not only form in normal numbers in triple knock-down embryos, but their gross morphology (cell soma size, shape and axon trajectory) is also unchanged even though the vast majority of these cells are no longer glycinergic or GABAergic (Figs. 4J–M and Supp. Data Table 3). Taken together, these observations suggest that the phenotype from knocking-down Pax2/8 is very specific and is not due to toxicity or to the cells dying.

To further confirm that our results were not caused by non-specific effects of the morpholino injections we also injected two different morpholinos at the same total concentration (4.5 mg/ml) as our Pax2/8 triple knock-down experiments. These morpholinos were an *evx1* translation blocking morpholino: CCTTTCCGTGCTTCGGCGAGCCCAT and a *lis1* control morpholino: CTgGTaGCCTCTGTGACAGgACgAT (small case letters show mismatches) which was a kind gift from Richard Adams. We injected these morpholinos into both wild-type embryos and into *noi* (*pax2a*) mutant embryos. In all of these experiments there was no change in the number of glycinergic and GABAergic spinal neurons (Supp. Data Fig. 7).

In all of our experiments, we observed a continuum of results, presumably due to slight variations in the amount of knock-down achieved from the MOs in different embryos and in individual cells within injected embryos. Very occasional morphologically disturbed embryos were excluded from further analysis. Each experiment was repeated 3 times (∼ 60 embryos injected each time). Results are shown for the 4 most severely effected embryos from each experiment (12 embryos in total).

### Cell counts and statistical analyses

In all cases, cell counts are for both sides of a 5-somite length of spinal cord adjacent to somites 6–10. Cell row numbers are assigned ventral to dorsal (e.g. cells directly above the notochord are in row 1, see Supp. Data Fig. 1F). The Pax2-expressing spinal cord domain is defined as rows 4–7 based on results shown in Fig. 1. Results were analysed using the students' *T* test. Statistically significant results where *p* < 0.05 are indicated with a star in the figures and individual *p* values are provided in Supp. Data Table 2. Error bars in figures indicate the standard deviation. Results in the text are shown as the mean ± standard deviation. For all of the knock-down experiments (morpholino injections and analysis of *noi* mutants) both the experimental and control results are an average of 12 embryos. For analyses that only include wild-types (e.g. determining the neurotransmitter phenotypes of Pax2-expressing cells in wild-type embryos) results are an average of 5 different embryos.

## Results

### Pax2/8-expressing zebrafish interneurons are predominantly glycinergic and/or GABAergic

In 24 h zebrafish embryos, *pax2a*, *pax2b* and *pax8* all have very similar expression patterns, suggesting that they are co-expressed by several distinct interneuron populations that form initially in the intermediate region of the spinal cord (Figs. 1A–C, E, I and J; Pfeffer et al., 1998). Consistent with this, similar numbers of cells are labelled by *in situ* hybridisation in wild-type embryos when *pax2a*, *pax2b* or *pax8* probes are hybridised either singly or together (Figs. 1A–C, E and I) and 2-colour double *in situ* hybridisation for *pax2a* and *pax2b* shows that these two genes are indeed expressed by the same spinal cord cells (Fig. 1J). Pax2 and Pax8 are part of a subfamily of Pax transcription factors that also includes Pax5 (Bouchard et al., 2000, 2002; Hans et al., 2004; Holland et al., 2007; Pfeffer et al., 1998; Wada et al., 1998; Walther et al., 1991). However, *pax5* is not expressed in the zebrafish spinal cord (Fig. 1H; http://www.zfin.org; Pfeffer et al., 1998).

To determine the neurotransmitter phenotypes of zebrafish *pax2*-expressing interneurons we combined immunohistochemistry for Pax2 and *in situ* hybridisation for markers specific for glycinergic, GABAergic and glutamatergic cells (see Materials and methods). At 24 h, about 90% of Pax2-expressing spinal cells are either glycinergic and/or GABAergic, with ∼ 60% being glycinergic and a similar percentage GABAergic (Figs. 2A, B, D and E; 89.2% ± 4.7 express either GABAergic or glycinergic markers, 57.6% ± 6.5 are glycinergic and 57.1% ± 3.6 are GABAergic; see also Supp. Data. Fig. 2). This suggests that about a third of Pax2-expressing spinal cells express both GABAergic and glycinergic markers at this stage. Intriguingly a small number of Pax2-expressing spinal cells express glutamatergic markers (Figs. 2C and E; 16.4% ± 1.6). These cells are predominantly in the most dorsal row of Pax2 expression where they appear to correspond to the larger cells that are the first cells in the spinal cord to express Pax2 (Mikkola et al., 1992).

Pax2-expressing interneurons account for ∼ 80% of the glycinergic and ∼ 34% of the GABAergic interneurons in the zebrafish spinal cord (Figs. 2A, B and F; 80.4% ± 6.6 and 33.8% ± 2.9 respectively). However, most of the GABAergic cells that do not express Pax2 are found in the ventral spinal cord outside the Pax2-expression domain (Figs. 2B and cf 2F and 2G). Within the intermediate spinal cord region where Pax2 is expressed (rows 4–7), ∼ 75% of GABAergic cells express Pax2 (Fig. 2G; 74.8% ± 11.7). This shows that not only are the vast majority of Pax2-expressing spinal cells glycinergic and/or GABAergic, but in the spinal cord region where Pax2 is expressed, most glycinergic and GABAergic neurons express Pax2.

### Pax2/8 act redundantly to specify glycinergic and GABAergic fates of multiple interneurons in zebrafish spinal cord

To determine whether Pax2 is required for glycinergic and/or GABAergic fates in the spinal cord, we first examined zebrafish embryos that lack Pax2a function (*no isthmus* (*noi*) mutants) at 24 h (Brand et al., 1996; Lun and Brand, 1998). In all cases, loss of Pax2a had no effect on the number of cells with particular neurotransmitter phenotypes (Figs. 3M and N; Supp. Data Fig. 3 and Supp. Data Tables 1 and 2). To test whether this is due to functional redundancy between Pax2a and Pax2b, we knocked-down Pax2b function in *noi* mutants using a *pax2b* morpholino (MO). At 24 h, we observed a statistically significant reduction in the number of glycinergic cells (Fig. 3M and Supp. Data Tables 1 and 2) showing that Pax2 function is required for the glycinergic fates of some spinal cord cells. However, there was no significant change in the number of GABAergic cells (Fig. 3N and Supp. Data Tables 1 and 2) and the reduction in the number of glycinergic cells was far less than the number of glycinergic interneurons that express Pax2 (cf. Figs. 3M and 2G).

Therefore, we tested whether there was functional redundancy between Pax2 and Pax8 by injecting MOs against *pax2b* and *pax8* into *noi* mutants. These triple knock-down embryos lost expression of *pax8* RNA (Fig. 1F) and Pax2 protein (Fig. 3I), suggesting that Pax2a, Pax2b and Pax8 were all fully knocked-down. We also confirmed that *pax5* expression was not activated in the spinal cord of these triple knock-down embryos (Fig. 1L). Interestingly, our results show that *pax8* spinal cord expression is regulated by Pax2 and may also be regulated by Pax8 (*pax8* expression is dramatically downregulated in the absence of either Pax2 or Pax8 function and it is completely lost in triple knock-down embryos; see Fig. 1F, Supp. Data Figs. 1C–E and discussion in Supp. Data Fig. 1 legend). However, in contrast *pax2* expression does not require Pax2/8 function (Figs. 1D and K).

In these 24 h triple knock-down embryos, we observed a dramatic decrease in the number of glycinergic and GABAergic spinal cord cells in the intermediate region of the dorso-ventral axis where Pax2 is normally expressed (Figs. 3D, E and M–P). In contrast, wild-type embryos injected with *pax8* MOs had only minor reductions in the number of glycinergic and GABAergic cells (Supp. Data Fig. 3 and Supp. Data Tables 1 and 2), supporting the hypothesis that *pax2a*, *pax2b* and *pax8* function redundantly in specifying glycinergic and GABAergic fates.

We confirmed that our morpholino injections were not causing either specific or non-specific cell death by demonstrating that the same number of cells express *pax2* RNA in wild-type and triple knock-down embryos (Figs. 1C, D and K), that there was no upregulation of *p53* (Robu et al., 2007) in triple knock-down embryos (Supp. Data Fig. 1H) and that injection of two different control morpholinos into wild-type or *noi* (*pax2a*) mutants had no effect on glycinergic or GABAergic spinal neurons (Supp. Data Fig. 7; see also the longer discussion of morpholino control experiments in Materials and methods). In addition, we demonstrated that the reduction in the number of glycinergic and GABAergic cells is not caused by cells changing to a glutamatergic or acetylcholinergic fate as wild-type and triple knock-down embryos have the same number of glutamatergic and acetylcholinergic spinal cord cells (Figs. 3C, F, J–L and Q).

**Fig. 5:**
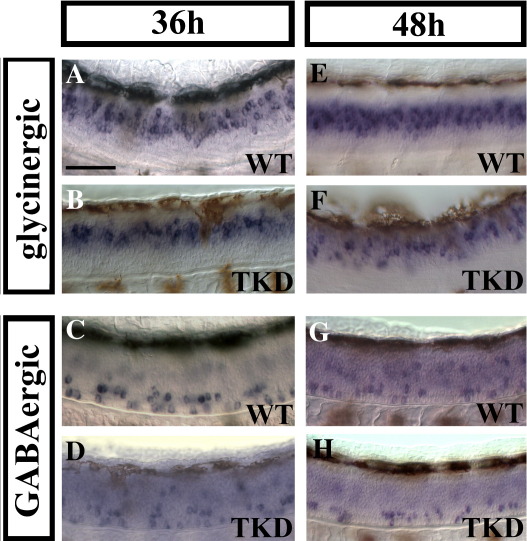
Triple knock-down embryos still have reduced numbers of glycinergic and GABAergic spinal neurons at 36 h and 48 h. Lateral views of *in situ* hybridisation for markers of glycinergic (A, B, E and F) and GABAergic (C, D, G and H) cell fates in wild-type (WT; A, C, E and G) and triple knock-down (TKD; B, D, F and H) trunks at 36 h (A–D) and 48 h (E–H). (Cell counts for this data are shown inSupp. Data Figs. 4A and A'.) Brown cells are melanophores that have differentiated and started to migrate by these stages. Rostal is left, dorsal is top. Scale bar = 50 μm.

Finally, to confirm that glycinergic and GABAergic fates are not just delayed in triple knock-down embryos, we also examined wild-type and triple knock-down embryos at 36 h and 48 h. In both cases, we still observed a significant reduction in the number of glycinergic and GABAergic neurons in triple knock-down embryos compared to stage-matched wild-type controls (Supp. Data Figs. 4A and A' and Supp. Data Fig. 5).

As our results are consistent with those previously reported by other researchers in the dorsal horn of the mouse *Pax2* knock-out (Cheng et al., 2004; Pillai et al., 2007), it seems very unlikely that the phenotypes we observe in triple knock-down embryos are due to off-target effects from the morpholinos (i.e. to one of the morpholinos knocking-down a different gene, that is required for glycinergic or GABAergic fates). However, to further confirm this, we examined all of the different combinations of Pax2/8 knock-down (Supp. Data Fig. 3 and Supp. Data Table 1 and Table 2). Two observations argue strongly that loss of glycinergic and GABAergic spinal fates is specifically caused by knocking-down Pax2/8 function. Firstly, we see a continuum of phenotypes depending on how many Pax2/8 proteins we knock-down, regardless of whether we use the *pax2b* MO, the *pax8* MOs or the *noi* (*pax2a*) mutants (Supp. Data Fig. 3 and Supp. Data Table 1 and Table 2). Secondly, we observe a statistically significant difference between wild-type embryos injected with *pax8* and *pax2b* morpholinos and *noi* mutants injected with *pax8* and *pax2b* morpholinos (the triple knock-downs are always more severely affected than the double knock-downs, *p* = 0.005 in the case of glycinergic cells and *p* = 0.0045 in the case of GABAergic cells; see also Supp. Data Fig. 3 and Supp. Data Table 1 and 2). If our results were due to off-target effects from the morpholinos then this would not be the case. We would instead expect these two results to be very similar to each other.

Taken together, our results demonstrate that Pax2/8 are redundantly required for the glycinergic and/or GABAergic fates of many zebrafish spinal cord interneurons, but that the absence of Pax2/8 function (and of a glycinergic and/or GABAergic fate) is not sufficient for cells to become glutamatergic or cholinergic.

### Pax2/8 are required to specify glycinergic fates of most CiAs

In mouse, Pax2 is required for the neurotransmitter fates of dorsal horn GABAergic neurons but it is not required for the neurotransmitter fates of more ventral Pax2-expressing interneurons (Cheng et al., 2004; Pillai et al., 2007). In contrast, our results suggest that Pax2/8 are required for GABAergic and glutamatergic fates throughout the dorsal–ventral extent of the Pax2-expression domain. To further confirm this, we specifically examined the most ventral population of Pax2-expressing spinal cells. In amniotes, these are V1 cells. In zebrafish, Circumferential Ascending interneurons (CiAs) (Bernhardt et al., 1990) are thought to be homologous to V1 cells (Higashijima et al., 2004b; Sapir et al., 2004). Both V1 cells and CiAs are the only spinal cells to express the transcription factor Eng1b (in the case of zebrafish) or En1 (in the case of amniotes) (Higashijima et al., 2004b; Sapir et al., 2004). In addition, both of these cell types share morphological and functional characteristics. For example, both CiAs and V1 cells are inhibitory, they have ipsilateral ascending axons and they are involved in regulating fast locomotion movements (Gosgnach et al., 2006; Higashijima et al., 2004b; Li et al., 2004). However, before this study, it had not been determined whether CiAs also, like V1 cells, express Pax2.

**Fig. 6:**
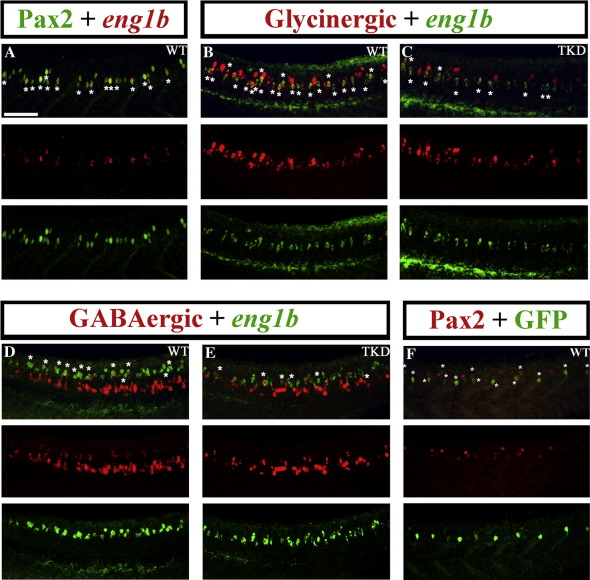
Lateral views of wild-type (WT; A, B and D) triple-knock-down (TKD; C and E) and Tg(*pax2a:*GFP) (F) trunks at 24 h. Rostal is left, dorsal is top. (A) Pax2 (green), *eng1b* (red). (B–E) Double *in situ* hybridisation for *eng1b* (green) and neurotransmitter markers (red). (F) Pax2 (red), GFP (green). The pictures in this figure are the same as inFig. 2 in the main paper — but this supplementary data figure also shows single channel images for each result (and panel F in this figure was panel I inFig. 4 in the main paper). Stars in merged images indicate double labelled cells. Scale bar = 50 μm.

**Fig. 7:**
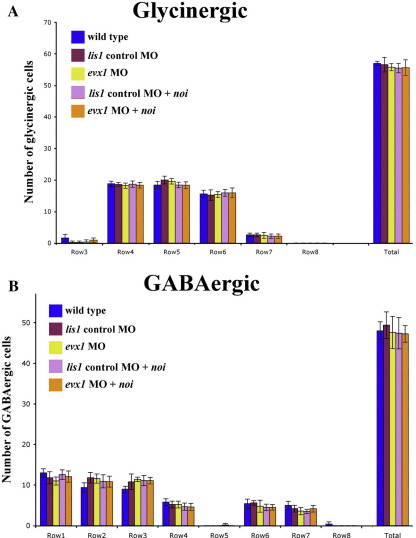
Control morpholinos have no effect on inhibitory neuronal fates. Graphs show the number of glycinergic (A) and GABAergic (B) cells at 24 h in wild-type embryos, wild-type embryos injected with *evx1* or *lis1* control morpholinos at a concentration of 4.5 mg/ml and *noi* (*pax2a*) mutant embryos injected with *evx1* or *lis1* control morpholinos at a concentration of 4.5 mg/ml. Cell counts are for a 5 somite length of the spinal cord adjacent to somites 6–10, broken down row by row and shown (RHS) as a total. Rows not shown have a cell count of zero for all 5 conditions. Error bars denote standard deviation. All numbers are an average from 5 different embryos.

Using *in situ* hybridisation for *eng1b* and immunohistochemistry for Pax2 we demonstrated that at 24 h CiAs express Pax2 and that they are indeed the most ventral spinal cord cells to do so (Fig. 4A; see also Supp. Data Fig. 6A). Consistent with this, we also showed that 24 h embryos from a transgenic line where GFP is regulated by a partial *pax2* promoter (Picker et al., 2002), express GFP in a subset of spinal cord Pax2-expressing cells, the majority of which are CiAs (Figs. 4F and I–M).

Determining that CiAs express Pax2 provided us with the opportunity not only to investigate the effects of Pax2/8 knock-down on the most ventral population of Pax2/8-expressing spinal neurons, but also to examine the morphology and neurotransmitter phenotypes of a single identified class of neurons. Our more global analysis of triple knock-down embryos identified a dramatic decrease in the number of glycinergic and GABAergic cells in the intermediate region of the spinal cord where Pax2/8 are normally expressed (Figs. 3D, E and M–P). However, some glycinergic and GABAergic cells still remain in these triple knock-down embryos and it was unclear whether this was due to incomplete penetrance of the phenotype or due to Pax2/8 only being required for the glycinergic and/or GABAergic fates of specific subsets of Pax2/8-expressing neurons. Determining the phenotype of CiAs in triple knock-down embryos should enable us to distinguish between these two possibilities. In the former case, we would expect some CiAs to maintain their glycinergic and/or GABAergic fates in triple knock-down embryos, whereas in the latter case we would expect all CiAs to have the same phenotype (either loss of glycinergic and GABAergic fates or no effect).

At 24 h, ∼ 90% of CiAs are glycinergic and just over 40% are GABAergic, suggesting that several CiAs express both of these neurotransmitters at this stage (Figs. 4B, D and N; 89.52% ± 6.87 of CiAs are glycinergic and 42.59% ± 5.04 are GABAergic; see also Supp. Data Fig. 6 and Higashijima et al., 2004b). Consistent with our analyses of whole spinal cords, in triple knock-down embryos the number of CiAs (*eng1b*-expressing cells) is not altered, but the number of glycinergic and GABAergic CiAs is reduced (Figs. 4C, E and N; in triple knock-down embryos only 23.52% ± 5.43 of CiAs are glycinergic and 27.69% ± 6.86 are GABAergic; see also Supp. Data Fig. 6). However, no CiAs are glutamatergic, in either wild-type or triple knock-down embryos (Figs. 4G and H) and the general size and shape of CiA somata and CiA axon lengths and trajectories are indistinguishable in wild-type and triple knock-down embryos (Figs. 4J–M and Supp. Data Table 3). This suggests that the loss of glycinergic and GABAergic fates is a very specific phenotype and that in other respects these cells develop normally (at least at these early stages).

These results also suggest that the incomplete penetrance of the Pax2/8 knock-down phenotype is not due to specific Pax2/8-expressing populations being resistant to loss of Pax2/8 function. CiAs are thought to constitute a single class of neurons (Higashijima et al., 2004b), but in triple knock-down embryos some CiAs still maintain their glycinergic and GABAergic fates.

## Discussion

### Pax2/8 act redundantly to specify glycinergic and/or GABAergic fates of many spinal cord interneurons

In this paper, we provide the first systematic analysis of the neurotransmitter fates of all Pax2-expressing spinal interneurons. We show that in zebrafish embryos, the vast majority of Pax2-expressing interneurons are glycinergic or GABAergic and these cells account for ∼ 60% of all glycinergic and GABAergic spinal interneurons and ∼ 86% of glycinergic and GABAergic interneurons within the spinal cord regions where Pax2 is expressed. Studies of specific subsets of Pax2-expressing spinal cells in mouse have demonstrated that many of these neurons are also GABAergic and/or glycinergic (Cheng et al., 2004; Lewis, 2006 and references therein; Pillai et al., 2007; Sapir et al., 2004), suggesting that this correlation of Pax2 expression and glycinergic and GABAergic fates is highly conserved among vertebrates.

We also show that Pax2a, Pax2b and Pax8 act in a functionally redundant manner to specify the glycinergic and GABAergic fates of many Pax2/8-expressing spinal interneurons. When we knock-down Pax2a, Pax2b and Pax8 many interneurons lose their glycinergic and GABAergic fates, including the majority of CiAs, which are the most ventral population of Pax2/8-expressing spinal cells. We also establish that this function of Pax2/8 in spinal interneurons is very specific: loss of Pax2/8 function does not cause cells to change their neurotransmitter phenotype and become excitatory (glutamatergic or cholinergic); they are just no longer glycinergic or GABAergic. In addition, at least in the case of CiAs, their soma shapes and sizes and axon trajectories are unchanged. It is still formally possible that these neurotransmitter phenotypes are an indirect effect caused by a lack of synapse formation and/or synaptic activity in these neurons. However, given that we observe a dramatic neurotransmitter phenotype as early as 24 h we consider that this is unlikely.

Our results suggest that the lack of a neurotransmitter phenotype in ventral Pax2-expressing cells in the mouse Pax2 knock-out, is at least partly due to redundancy between Pax2 and Pax8. Consistent with this, in *Pax2* mutant mice Pax8 continues to be expressed in the ventral spinal cord, but it is lost from the Pax2-expressing cells that migrate into the dorsal horn (Pillai et al., 2007). This suggests that Pax2/8 have a major and crucial function in specifying glycinergic and GABAergic fates of multiple spinal cord interneurons in both the simple anamniote and the more complex mammalian spinal cord.

As mentioned earlier, Pax2 and Pax8 are part of a subfamily of Pax transcription factors that also includes Pax5 (Bouchard et al., 2000, 2002; Hans et al., 2004; Holland et al., 2007; Pfeffer et al., 1998; Wada et al., 1998; Walther et al., 1991). Unlike in zebrafish, in mouse Pax5 is expressed in the spinal cord (Pillai et al., 2007), raising the possibility that it may also function redundantly with Pax2 and Pax8 in mammalian spinal cord.

### Other factors must also be involved in specifying glycinergic and GABAergic spinal fates

While knock-down of Pax2a, Pax2b and Pax8 in zebrafish embryos results in substantial and statistically significant reductions in the number of spinal interneurons with glycinergic and/or GABAergic fates, several glycinergic and/or GABAergic spinal interneurons remain in these triple knock-down embryos. Many of the remaining GABAergic neurons are located in the very ventral spinal cord (rows 1–3; Fig. 3E) outside the Pax2/8 expression domain (rows 4–7: Fig. 1I) and, hence, these GABAergic neurons must be specified by a different mechanism. However, in addition, a minority of Pax2/8-expressing cells retain their glycinergic and/or GABAergic fates in triple knock-down embryos. Interestingly, this is the case even for CiAs, which are thought to constitute a single class of neurons (Higashijima et al., 2004b). Furthermore, in triple knock-down embryos the number of glycinergic cells is reduced much more dramatically than the number of GABAergic cells. This might suggest that Pax2/8 have a more pronounced role in specifying glycinergic neurons than GABAergic neurons. Alternatively Pax2/8 might be required for maintenance of glycinergic and GABAergic fates rather than their initial specification, as CiAs (and potentially other zebrafish spinal neurons) change from a GABAergic to a glycinergic fate during their development (Higashijima et al., 2004b). However, in this case we would expect there to be a more severe reduction of glycinergic and/or GABAergic neurons in triple knock-down embryos at later stages, but this is not what we observe (Supp. Data Figs. 4A and A' and Supp. Data Fig. 5).

One possible explanation for the phenotype not being completely penetrant might be an incomplete knock-down of Pax2/8 function. However, in this case any remaining Pax2/8 expression must be very weak as it is not detected by Pax2 immunohistochemistry or *pax8 in situ* hybridisation (Figs. 1F and 3I). Therefore, we think that it is more likely that, while Pax2/8 are major players in specifying glycinergic and GABAergic spinal fates, there are other factors that can compensate for the loss of Pax2/8 in some cells. For example, it is possible that Pax2/8 may only be required for glycinergic and/or GABAergic neurotransmitter expression in as-yet-unidentified distinct subsets of CiAs and other Pax2/8-expressing cells. Alternatively, induction of glycinergic and GABAergic fates may be a strongly buffered mechanism where other transcription factors can sometimes, in a stochastic manner, substitute for loss of Pax2/8. If this is the case, then it is not yet clear what these additional, as-yet-unidentified, transcription factors might be. In the mouse dorsal horn, Lbx1, Ptf1a, Lhx1 and Lhx5 transcription factors are also required for correct specification of GABAergic fates. However, all of these proteins act upstream of Pax2 and control neurotransmitter fates by regulating *pax2* expression (Cheng et al., 2004, 2005; Glasgow et al., 2005; Pillai et al., 2007) so they are unlikely to compensate for loss of Pax2/8.

Finally, another possible explanation for some Pax2/8-expressing glycinergic and GABAergic neurons maintaining their neurotransmitter fates is suggested by the observation that spontaneous neuronal activity can homeostatically bias the specification of excitatory versus inhibitory spinal fates. Experiments in frogs have shown that spinal cord neurons with particular neurotransmitter fates share specific patterns of calcium spiking during their early development. If these calcium spikes are blocked by genetic or pharamacological agents then the number of inhibitory neurons decreases and the number of excitatory neurons increases, whereas the opposite phenotype is observed if calcium activity levels are increased (Borodinsky et al., 2004; Spitzer et al., 2004). Interestingly, the genetic identities of these cells are unchanged, suggesting that activity levels can, in some instances, over-ride other developmental cues that specify neurotransmitter fates. Spontaneous intracellular calcium signals have also been observed during early development of zebrafish spinal cord neurons (Ashworth and Bolsover, 2002). Therefore, the incomplete loss of glycinergic and GABAergic fates in neurons that normally express Pax2/8 in triple knock-down embryos could be due to some glycinergic and GABAergic fates being maintained by this activity-based mechanism. However, it is not yet clear whether this mechanism acts upstream, downstream or in parallel to transcription factor specification of neurotransmitter fates. In addition, in these experiments neurons either switched their fates (from inhibitory to excitatory or vice versa) or, in a small number of cases, they co-expressed excitatory and inhibitory neurotransmitters (Borodinsky et al., 2004), whereas in Pax2/8 triple knock-down experiments many neurons lose their glycinergic and GABAergic fates, but they do not acquire glutamatergic or cholinergic fates.

### Pax2/8 are not sufficient to specify glycinergic or GABAergic fates

While about 90% of Pax2/8-expressing spinal interneurons are either glycinergic or GABAergic at 24 h, only ∼ 60% are glycinergic and ∼ 60% are GABAergic. In addition, a minority of Pax2/8-expressing cells in both zebrafish and mouse dorsal spinal cords express glutamatergic markers (Figs. 2C and E; Cheng et al., 2004). This suggests that while Pax2/8 are required for glycinergic and GABAergic fates in many cells, expression of these transcription factors is not sufficient to specify one or other of these neurotransmitter fates. This is consistent with data reported in chick, where ectopic expression of Pax2 in the neural tube did not induce GABA expression (Cheng et al., 2004).

It is not clear from our results why some Pax2/8-expressing cells are glycinergic, some are GABAergic and some express both of these neurotransmitters. It is possible that at least some of the GABAergic cells or the cells expressing both GABA and glycine will later become glycinergic as has been suggested for CiAs (Higashijima et al., 2004b). Therefore, at least some of these differences in neurotransmitter fates may reflect a temporal progression within particular neurons from (GABAergic) to (GABAergic and glycinergic) to (glycinergic). Consistent with this, the number of glycinergic neurons more than doubles between 24 h and 48 h whereas the number of GABAergic neurons stays pretty much constant (Supp. Data. Figs. 4A and A'). However, it is also possible that in at least some cases, these different neurotransmitter fates reflect more profound differences between cells. In this case, these differences may result from differential expression of other transcription factors or different developmental histories.

The simplest explanation for the Pax2/8-expressing glutamatergic cells would be that there is another transcription factor expressed in these cells that inhibits the function of Pax2/8 and instead specifies a glutamatergic phenotype. There is a precedence for this type of mechanism, in that the Lbx1 transcription factor normally specifies GABAergic interneurons in the dorsal spinal cord, but in a subset of Lbx1-expressing cells, Tlx3 inhibits the function of Lbx1 and induces a glutamatergic phenotype (Cheng et al., 2005).

### Specificity of the Pax2/8 triple knock-down phenotype

One of the potentially surprising aspects of our results is the specificity of the phenotype that we observe in the absence of Pax2/8 function. While we cannot rule out that additional phenotypes develop at later stages in the neurons that normally express Pax2/8 in triple knock-down embryos (and it would be hard to test this using morpholinos as their efficacy decreases with increasing age of the embryos and dilution of the morpholinos), the only phenotype that we have identified so far in these neurons is a loss of glycinergic and GABAergic fates. This might initially be surprising, given that, as discussed above, neuronal activity can homeostatically adjust neurotransmitter fates in the spinal cord (Borodinsky et al., 2004) and given a recent report that knocking-down glycine receptors reduces the number of neurons (including Pax2-expressing neurons) in the zebrafish spinal cord (McDearmid et al., 2006). However, with respect to the former study, the specification of neurotransmitter fates by calcium activity is thought to be cell autonomous (so defects in a subset of spinal neurons shouldn't affect other neurons) and in the case of the latter study, decreases in neuronal numbers were only observed at later stages of development. While neuronal morphology was not examined in the mouse *Pax2* mutant dorsal horn, these studies also observed that the cells that lost GABAergic fates did not become glutamatergic, suggesting that the specificity of the *Pax2/8* phenotype may be highly conserved between different vertebrates.

### Functional consequences of loss of Pax2/8

In zebrafish, the only Pax2/8-expressing interneurons that have been identified morphologically or functionally are CiAs (this report). These are genetically and functionally homologous to mammalian V1 cells (Alvarez et al., 2005; Higashijima et al., 2004b; Sapir et al., 2004; Saueressig et al., 1999). However, comparisons with amniotes suggest that more dorsal Pax2/8-expressing interneurons in the zebrafish spinal cord are likely to be functionally equivalent to amniote V0, dI6, dI4 and/or DIL_A_ interneurons (Lewis, 2006 and references therein). Of these neurons, DIL_A_ neurons migrate into the superficial lamina of the dorsal horn where they are probably involved in nociception, dI4 cells are thought to migrate into deeper layers of the dorsal horn and V1, V0 and dI6 interneurons form part of the locomotion central pattern generator in the ventral spinal cord. At least a subset of V0 interneurons are essential in mice for correct walking movements as they control the alternating left–right activity of the motor neurons that innervate hindlimb muscles (Lanuza et al., 2004). In contrast, V1 cells are required for fast locomotion in mouse: if these cells are genetically ablated or temporarily inactivated, mice are unable to walk at fast speeds but they can still walk at slower speeds (Gosgnach et al., 2006). This is also consistent with data from tadpoles, which shows that aINs (which are genetically homologous to both CiAs and V1 cells) provide early cycle inhibition to central pattern generator neurons during swimming, particularly at faster swimming frequencies (Li et al., 2004). Based on these results in other vertebrates, we would predict that altering the neurotransmitter phenotypes of Pax2/8-expressing cells might have functional consequences both for sensory processing and correct locomotion.

Unfortunately, this is not easy to assess in our experiments, as we can't distinguish between behavioural defects due to the spinal cord phenotype that we describe in this paper and behavioural defects due to previously described brain phenotypes (for example even *noi* single mutant embryos lack a midbrain–hindbrain boundary";; Brand et al., 1996; Lun and Brand, 1998). However, we do observe considerable locomotion defects in triple knock-down embryos. For example, triple knock-down embryos only move when they are poked (even at stages where wild-type embryos undergo spontaneous fast swimming movements), a significant percentage of triple knock-down embryos don't move at all (5/20 at 24 h, 3/20 at 48 h) and an even larger percentage swim in circles (8/20 at 48 h), or vibrate on the spot (6/20 at 48 h) when poked. All of this is at least consistent with the idea that locomotion control is perturbed in these embryos.

## Conclusions

Taken together, all of these results suggest that Pax2/8 transcription factors have crucial but redundant functions in specifying the glycinergic and GABAergic fates of multiple spinal interneurons in both the simple anamniote and the more complex mammalian spinal cord. Comparisons between zebrafish, *Xenopus* and mouse have suggested that mechanisms of spinal cord patterning and resulting neuronal circuitry might be highly conserved in vertebrates, with distinct functional classes of interneurons being specified by different combinations of post-mitotically expressed transcription factors (Goulding and Pfaff, 2005; Lewis, 2006). However, to our knowledge, our study is the first that has demonstrated that some of these post-mitotically expressed transcription factors, do indeed have similar functions in anamniote and mammalian spinal cord interneurons.

## Figures and Tables

**Fig. 1:**
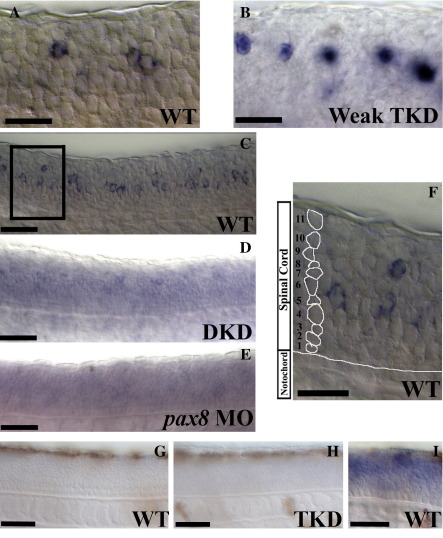
Lateral views of *pax8* expression in 24 h trunks (A–F) and *p53* expression in 36 h trunks (G–I). *pax8* morpholinos block the splicing of *pax8* RNA causing it to be retained in the nucleus (see discussion of splice-blocking morpholinos inYan et al., 2002). (A) Cytoplasmic *pax8* RNA in wild-type embryos; (B) unspliced nuclear *pax8* RNA in *noi (pax2a)* mutants injected with 1 mg/ml each of *pax2b* and *pax8* MOs (“weak” triple knock-down (TKD) embryos). When these morpholinos are injected at a higher concentration, expression of *pax8* is lost (seeFig. 1F in the main paper). *pax8* expression depends at least partly on Pax2 function. In *noi* mutants injected with 1.5 mg/ml *pax2b* MO (double knock-down (DKD) embryos), the expression of *pax8* is dramatically reduced (D and cf. C). While *pax8* expression is very weak in these embryos, it is more substantial than in triple-knock-down (TKD) embryos (cf.Fig. 1F in the main paper) suggesting either that Pax8 acts redundantly with Pax2 in regulating its own expression and/or that any remaining *pax8* RNA in TKD embryos is degraded (probably because the *pax8* splice-blocking MOs have prevented it from being processed properly). In support of the latter hypothesis, *pax8* expression is almost completely lost in wild-type embryos injected with *pax8* morpholino alone (E), although this could also indicate that Pax8 function is required for *pax8* expression in the spinal cord. (F) is a higher magnification of the box in (C) demonstrating how cell rows were counted. For each positive cell, the number of cells between it and the notochord were counted to determine which row it was in. Example row numbers are illustrated for the column of cells on the left of the picture. *p53* expression is not activated in TKD embryos. (G) shows that *p53* is not expressed in 36 h WT trunks and (H) shows that this is also the case in 36 h TKD trunks. (I) is a control to show that the *in situ* hybridisation is working: this photograph shows *p53* expression in a damaged 36 h WT embryo. Scale bars = 25 μm (A, B and F) and 50 μm (C–E and G–I).

**Fig. 2:**
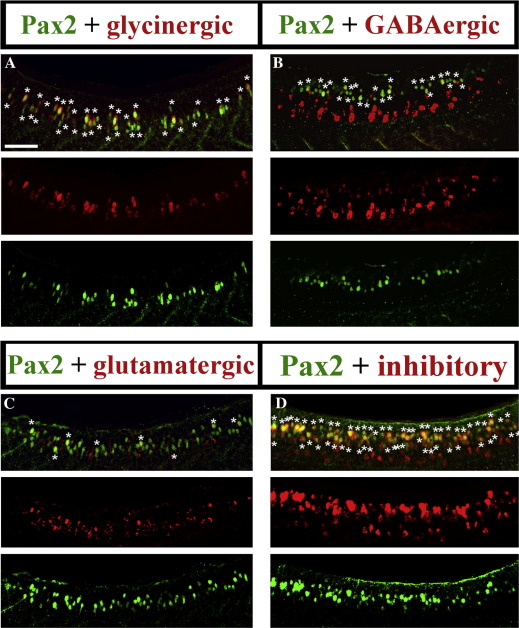
Lateral views of Pax2 immunohistochemistry and *in situ* hybridisation for markers of glycinergic (A), GABAergic (B) glutamatergic (C) and GABAergic or glycinergic (labelled “inhibitory”; probes were mixed) (D) cell fates in 24 h WT trunks. SeeMaterials and methods for details of probes used. The pictures in this figure are the same as inFig. 2 in the main paper — but this supplementary data figure also shows single channel images for each result. Stars in merged images indicate double labelled cells. Rostal is left, dorsal is top. Scale bar = 50 μm.

**Fig. 3:**
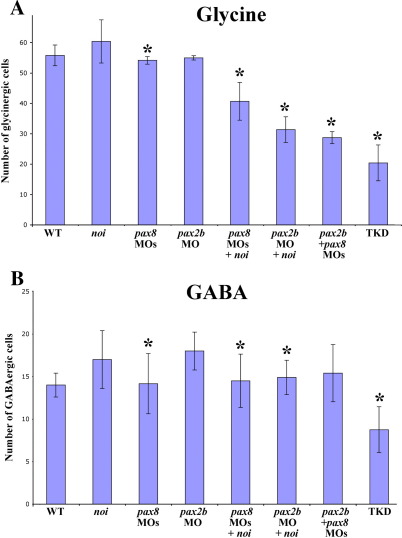
Number of glycinergic (A) and GABAergic (B) cells in the Pax2/8 expression domain (rows 4–7 of the spinal cord) in all of the different Pax2 and Pax8 knock-down experiments. Numerical values are given inSupp. Data. Table 1 and p values for all of the different pair wise comparisons are given inSupp. Data Table 2. In all cases, cell counts are for a 5 somite length of the spinal cord adjacent to somites 6–10. All values are an average from 12 different embryos. Error bars indicate the standard deviation. Results where the difference from wild-type is statistically significant are indicated with a star.

**Fig. 4:**
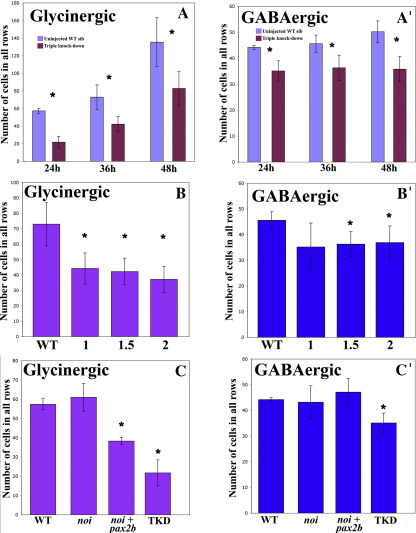
Glycinergic and GABAergic phenotypes of triple knock-down embryos were examined at different stages (A and A') and at different concentrations of morpholino injections (B and B'). Note that the severity of the phenotypes decreases slightly with age and increases slightly with the concentration of morpholinos injected. The data in A and A' was all obtained with morpholino concentrations of 1.5 mg/ml each; the data in B and B' was all obtained at 36 h. C and C' show the number of glycinergic and GABAergic cells throughout the dorsal/ventral extent of the spinal cord in different experimentally treated embryos. This data is taken from the same experiments asFigs. 3M and N in the main paper, but these graphs (C and C') show the number of cells in all rows, rather than just in the Pax2/8 expression domain. As inFigs. 1–4, cells were counted in a 5-somite length of zebrafish spinal cord adjacent to somites 6 to 10. Cell row numbers are counted ventral–dorsal from the most ventral part of the spinal cord (seeSupp. Data Fig. 1E). All numbers are an average from 12 different embryos. Stars indicate results that are statistically significant (p < 0.05). Error bars denote standard deviation. Photographs of the results shown in (A and A') are provided inSupp. Data Fig. 5.

**Fig. 1:**
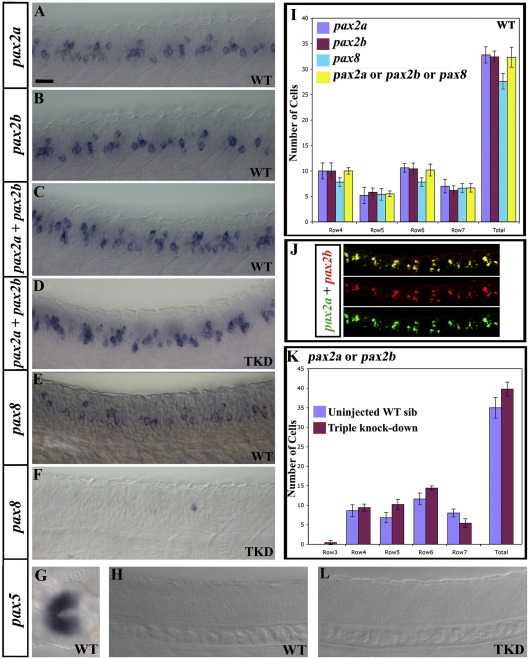
Expression of *pax2* and *pax8* genes in zebrafish spinal cord. *pax2a*, *pax2b* and *pax8* are co-expressed by many cells but *pax5* is not expressed in zebrafish spinal cord. Lateral views of *pax2a* (A), *pax2b* (B), *pax2a* and *pax2b* (C and D; probes were mixed), *pax8* (E and F), *pax5* (H and L) and *pax2a* and *pax2b* (J; *pax2a* in green and *pax2b* in red) expression in 24 h wild-type (WT) trunks (A–C, E and H) and triple-knock-down (TKD) embryos (*pax2a* mutants injected with *pax2b* and *pax8* MOs) (D, F and L). *pax8* staining is weaker than *pax2a* and *pax2b* and hard to photograph. This means that several weak *pax8*-expressing cells are hard to see in (E). However, when *pax8*-expressing cells were counted, similar numbers were obtained to when *pax2a*- or *pax2b*-expressing cells were counted (see I). (G) shows a dorsal view of the midbrain–hindbrain boundary of a 24 h wild-type embryo (a region of the embryo where *pax5* is expressed, to show that this *in situ* hybridisation probe works). (J) shows merged and single channel images of a 2 colour fluorescent double *in situ* with *pax2a* in green and *pax2b* in red. In all cases, rostral is left. In all cases except (G), dorsal is top. Scale bar = 25 μm (A–H and L); 18 μm (J). (I) Average number of cells expressing *pax2a*, *pax2b*, *pax8* and *pax2a*, *pax2b* or *pax8* (probes were mixed) at 24 h in 5 different WT embryos. As the number of cells expressing *pax2a*, *pax2b*, *pax8* or (*pax2a*, *pax2b* or *pax8*) are not statistically different from each other, these genes must be co-expressed in spinal cord cells. (K) Average number of cells expressing *pax2a* or *pax2b* (probes were mixed) in 12 uninjected *noi* sibs and 12 TKD embryos. In this and all other figures, cells were counted in a 5-somite length of spinal cord adjacent to somites 6–10. Cell row numbers are counted ventral to dorsal (e.g. cells directly above the notochord are in row 1, see Supp. Data Fig. 1F). Rows not shown have a cell count of zero. None of the differences in (I) and (K) are statistically significant. Error bars denote standard deviation.

**Fig. 2:**
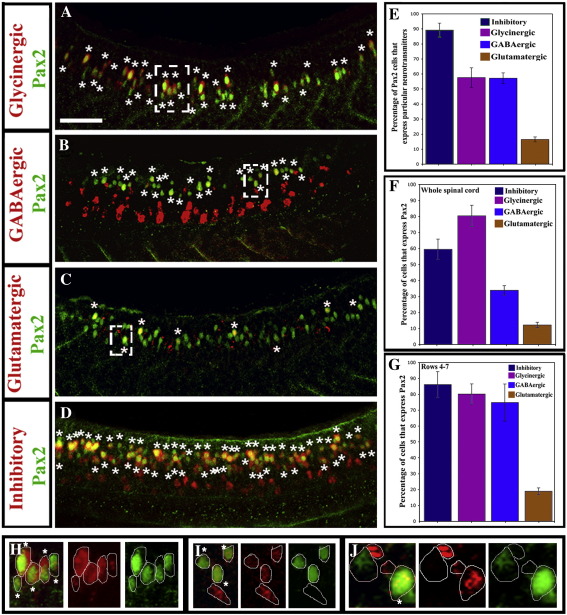
Most Pax2-expressing zebrafish spinal interneurons are inhibitory. Lateral views of Pax2 immunohistochemistry and *in situ* hybridisation for markers of glycinergic (A), GABAergic (B) glutamatergic (C) and GABAergic or glycinergic (labelled “inhibitory”; probes were mixed) (D) cell fates in 24 h WT trunks. See Materials and methods for details of probes used. Stars indicate double labelled cells. Rostal is left, dorsal is top. Scale bar = 50 μm. (E) Percentage of Pax2-expressing cells with particular neurotransmitter phenotypes. (F and G) Percentage of cells with specific neurotransmitter phenotypes that express Pax2 in whole spinal cord (F) or just the Pax2-expression domain (rows 4–7; G). In all cases, percentages are averages from 5 different embryos at 24 h. Error bars denote standard deviation. Note that most glycinergic cells are found in the Pax2/8 expression domain but many GABAergic cells are found more ventrally in the spinal cord (A and B; also cf. F and G). (H–J) Magnified views of dashed white boxes in A (H), B (I) and C (J) respectively showing red and green channels and merged images for a single confocal focal plane. Stars in merged images indicate double labelled cells. For single channel images of the whole lateral view see Supp. Data Fig. 2.

**Fig. 4:**
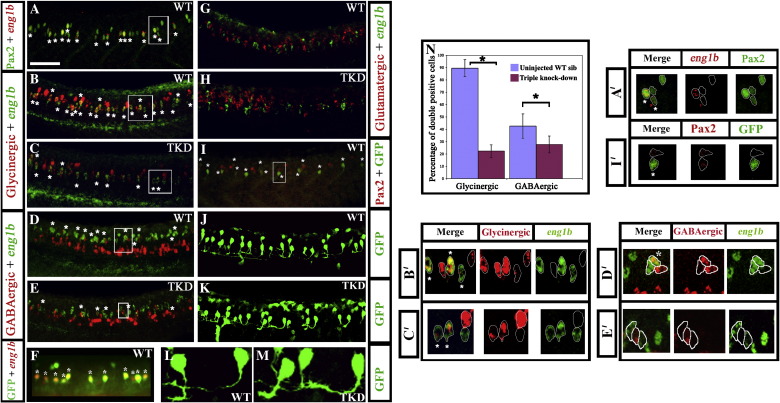
Pax2/8 specify inhibitory fates in CiA interneurons. Lateral views of 24 h trunks. (A, B, D and G) WT embryos, (C, E and H) triple-knock-down (TKD) embryos, (F, I, J and L) Tg(*pax2a*:GFP) WT embryos (K and M) Tg(*pax2a*:GFP) TKD embryos. Rostal is left, dorsal is top. (A) Pax2 (green), *eng1b* (red). (B–E, G and H) Double *in situ* hybridisation for *eng1b* (green) and neurotransmitter markers (red). *eng1b* staining is weaker in some pictures, but the same number of *eng1b*-expressing cells (13.9 ± 1.8 over a 5-somite length) were counted in each embryo (control and TKD). (F) Eng1b (red), GFP (green). The vast majority of GFP-positive cells in Tg (*pax2a*:GFP) embryos are CiAs and express Eng1b, although there are also a few more dorsal GFP-positive cells that are not CiAs. Occasionally a weak Eng1b-expressing cell can be observed that is not yet expressing GFP. (I) Pax2 (red), GFP (green). Note that all green cells are also red but several red cells are not green. (J–M) GFP (green). All of the ventral GFP-expressing cells are CiAs with similar ipsilateral ascending axons. (L and M) show higher magnification views of individual GFP-expressing CiAs in Tg(*pax2a*:GFP) WT (L) and TKD (M) embryos. For quantification of soma size and axon length see Supp. Data Table 3. Stars indicate double labelled cells. Scale bar = 50 μm (A–E and G–I); 30 μm (F, J and K). (N) Percentage of CiAs (*eng1b*-expressing cells) that are glycinergic or GABAergic in WT and TKD embryos. Each percentage is an average from 12 different embryos. Stars indicate statistically significant results (*p* < 0.05). Error bars denote standard deviation. No glutamatergic CiAs were observed in any embryo. (A'–E' and I') are magnified views of boxes in A–E and I respectively, showing red and green channels and merged images for a single confocal focal plane. Stars in merged images indicate double labelled cells. For single channel images of the whole lateral view for A–E and I see Supp. Data Fig. 6.

**Fig. 3:**
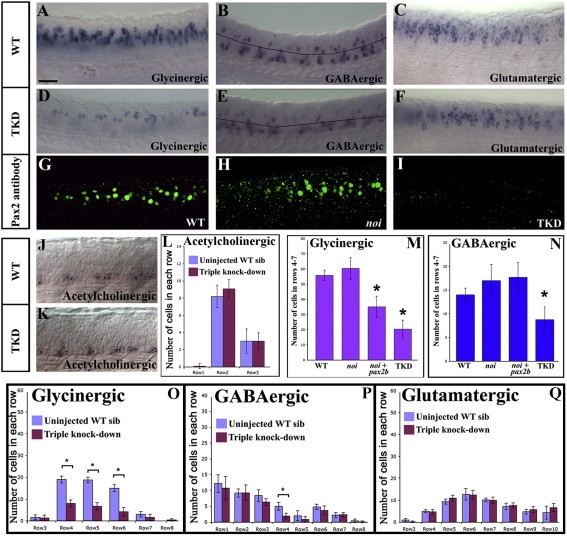
Pax2/8 act redundantly to specify inhibitory fates. Lateral views of *in situ* hybridisation for markers of glycinergic (A and D), GABAergic (B and E) glutamatergic (C and F) and acetylcholinergic (J and K) cell fates at 24 h in WT (A–C and J) and triple-knock-down (TKD) trunks (D–F and K). In (J and K) stars indicate cells expressing the acetylcholinergic marker, *chat*. In these photographs, some of the weaker cells are in a slightly different focal plane (on the other side of the spinal cord). In (B and E) the black line indicates the boundary between dorsal GABAergic cells within the Pax2/8 expression domain (rows 4–7) and more ventral GABAergic cells. Note that most remaining GABAergic cells in (E) are in the ventral region where Pax2/8 are not expressed and that none of the acetylcholinergic cells are within the Pax2/8 expression domain in either wild-type (J) or TKD (K) embryos. Rostal is left, dorsal is top. Scale bar = 40 μm. Immunohistochemistry for Pax2 in WT (G), *noi* (*pax2a*) mutant (H) and TKD (I) embryos. As Pax2 staining is present in (H) the antibody must recognise Pax2b. Therefore, lack of staining in (I) suggests that we have eliminated Pax2a + Pax2b protein in TKD embryos. (M and N) number of glycinergic and GABAergic cells at 24 h in Pax2/8 expression domain in WT, single-, double- and triple-knock-down embryos. Stars indicate statistically significant results (*p* < 0.05). For results in whole spinal cord see Supp. Data Figs. 4C and C'. For results of additional single and double knock-down experiments see Supp. Data Fig. 3 and Supp. Data Tables 1 and 2. (L and O–Q) show results for control and TKD embryos for all 4 neurotransmitters, broken down row by row. Rows not shown have a cell count of zero. In all cases, error bars denote standard deviation. The number of glycinergic and GABAergic cells is reduced in the Pax2/8 expression domain in TKD embryos but there is no significant change in the number of glutamatergic or acetylcholinergic cells in any region of the spinal cord.

## Appendix A Supplementary data

Table 1 Number of cells expressing particular neurotransmitters in the Pax2/8 expression domain (rows 4–7 of the spinal cord) in all of the different Pax2 and Pax8 knock-down experiments. In all cases, cell counts are for a 5 somite length of the spinal cord adjacent to somites 6–10. All values are an average from 12 different embryos and are shown as the mean + standard deviation. ND = not done.

Table 2 *P* values for all of the pair wise comparisons between the numbers of glycinergic or GABAergic cells in the different Pax2 and Pax8 knock-down experiments, calculated using the student *T* test. Results from experiments examining the number of glycinergic cells are in the left hand corner. Statistically significant results are shown in green (p < 0.05) and non-significant results are shown in blue. Results from experiments examining the number of GABAergic cells are in the right hand corner. Statistically significant results are shown in purple (p < 0.05) and non-significant results are shown in red. For example, the first box in the left hand column gives the *p* value for the hypothesis that *noi* mutants have the same number of glycinergic cells as wild-type embryos. As the *p* value < 0.05, the difference between the number of glycinergic cells in *noi* mutants and wild-type embryos is not statistically significant. In contrast, the bottom box in the left hand column gives the *p* value for the hypothesis that triple knock-down embryos have the same number of glycinergic cells as wild-type embryos. In this case, the *p* value for the comparison (and hence the difference between the number of glycinergic cells in triple knock-down embryos and wild-type embryos) is statistically significant.

Table 3 CiA axon length and soma size in wild-type and triple knock-down embryos at 24 h, measured using Zeiss Axiovision software. All of the measured CiAs were at the same rostral–caudal level (adjacent to somites 7 and 8). Values shown are averages + standard deviation. In the case of the WT results, the values are an average of 22 cells and in the case of the TKD results the values are an average of 19 cells. *p* values are for the student *T* test. Neither of the differences between WT and TKD CiAs is statistically significant.
