# Supplementary material for: Pax2/8 act redundantly to specify glycinergic and GABAergic fates of multiple spinal interneurons
Source: Dev Biol. 2008 Nov 1;323(1):88–97. doi: 10.1016/j.ydbio.2008.08.009 (PMC2849013; doi:10.1016/j.ydbio.2008.08.009)
Supplement: Table 1 — Number of cells expressing particular neurotransmitters in the Pax2/8 expression domain (rows 4–7 of the spinal cord) in all of the different Pax2 and Pax8 knock-down experiments. In all cases, cell counts are for a 5 somite length of the spinal cord adjacent to somites 6–10. All values are an average from 12 different embryos and are shown as the mean + standard deviation. ND = not done. [file mmc1.pdf]

Table 1. Number of cells expressing particular neurotransmitters in the Pax2/8 expression domain in different Pax2 and Pax8 knock-down experiments

Experiment	Glycinergic	GABAergic	Glutamatergic
**WT**	55.8 +/- 3.4	14 +/- 1.4	55+/-4.89
***noi* mutant**	60.4 +/- 7.1	17 +/- 3.4	ND
***pax2b* MO**	55 +/- 0.7	18 +/- 2.23	ND
***pax8* MOs**	54.17 +/- 1.25	14.16 +/- 3.54	ND
pax2b* MO + *noi	31.36 +/- 4.25	14.9 +/- 2.02	ND
***pax2b* + *pax8* MOs**	28.7 +/- 1.97	15.4 +/- 3.36	ND
pax8* MOs + *noi	40.67 +/- 6.2	14.5 +/- 3.13	ND
**TKD**	20.4 +/-5.9	8.75 +/- 2.7	58.42+/-5.40
