# Supplementary material for: Pax2/8 act redundantly to specify glycinergic and GABAergic fates of multiple spinal interneurons
Source: Dev Biol. 2008 Nov 1;323(1):88–97. doi: 10.1016/j.ydbio.2008.08.009 (PMC2849013; doi:10.1016/j.ydbio.2008.08.009)
Supplement: Table 2 — P values for all of the pair wise comparisons between the numbers of glycinergic or GABAergic cells in the different Pax2 and Pax8 knock-down experiments, calculated using the student T test. Results from experiments examining the number of glycinergic cells are in the left hand corner. Statistically significant results are shown in green (p < 0.05) and non-significant results are shown in blue. Results from experiments examining the number of GABAergic cells are in the right hand corner. Statistically significant results are shown in purple (p < 0.05) and non-significant results are shown in red. For example, the first box in the left hand column gives the p value for the hypothesis that noi mutants have the same number of glycinergic cells as wild-type embryos. As the p value < 0.05, the difference between the number of glycinergic cells in noi mutants and wild-type embryos is not statistically significant. In contrast, the bottom box in the left hand column gives the p value for the hypothesis that triple knock-down embryos have the same number of glycinergic cells as wild-type embryos. In this case, the p value for the comparison (and hence the difference between the number of glycinergic cells in triple knock-down embryos and wild-type embryos) is statistically significant. [file mmc2.pdf]

Table 2: p values for pair wise comparisons between different Pax2 and Pax8 knock-down experiments

GABAergic results

Expt	WT	*noi* mutant	*pax2b* MO	*pax8* MOs	*pax2b* MO + *noi*	*pax2b* + *pax8* MOs	*pax8* MO + noi	TKD
WT		0.10	0.724	0.031	0.00047	0.117	0.0035	1.36 x 10^{-8}
noi mutant	0.19		0.22	0.34	0.27	0.76	0.34	0.00050
pax2b MO	0.52	0.16		0.059	0.018	0.193	0.030	3.39 x 10^{-5}
pax8 MOs	0.049	0.149	0.627		0.879	0.569	0.85	0.0057
pax2b MO+ *noi*	3.29 x 10^{-10}	0.00027	1.435x10^{-9}	4.65 x 10^{-10}		0.57	0.94	1.96x10^{-6}
pax2b + *pax8* MOs	6.88 x 10^{-7}	0.00035	3.039x10^{-8}	1.81 x 10^{-9}	0.094		0.63	0.0045
pax8 MOs + *noi*	3.09 x 10^{-6}	0.0011	5.31x10^{-6}	5.2 x 10^{-6}	0.0004	2.28 x 10^{-5}		6.44 x 10^{-5}
TKD	2.0 x 10^{-10}	1.78 x 10^{-5}	2.24 x 10^{-10}	9.80 x 10^{-10}	0.0016	0.0005	1.32 x 10^{-7}	

Glycinergic results
