# Supplementary material for: Pax2/8 act redundantly to specify glycinergic and GABAergic fates of multiple spinal interneurons
Source: Dev Biol. 2008 Nov 1;323(1):88–97. doi: 10.1016/j.ydbio.2008.08.009 (PMC2849013; doi:10.1016/j.ydbio.2008.08.009)
Supplement: Table 3 — CiA axon length and soma size in wild-type and triple knock-down embryos at 24 h, measured using Zeiss Axiovision software. All of the measured CiAs were at the same rostral–caudal level (adjacent to somites 7 and 8). Values shown are averages + standard deviation. In the case of the WT results, the values are an average of 22 cells and in the case of the TKD results the values are an average of 19 cells. p values are for the student T test. Neither of the differences between WT and TKD CiAs is statistically significant. [file mmc3.pdf]

Table 3. CiA axon length and soma size in wild type and triple knock-down embryos

	**WT**	**TKD**	p value for the comparison
**Cell body area (μm^2)**	114.38 +/- 14.69	102.90 +/- 23.61	0.078
**Axon length (μm)**	85.57 +/- 17.78	81.17 +/-13.55	0.38
Number of cells examined	22 cells in 7 embryos	19 cells in 7 embryos	
